# A Difference of Past Self-Evaluation Between College Students With Low and High Socioeconomic Status: Evidence From Event-Related Potentials

**DOI:** 10.3389/fpsyg.2021.629283

**Published:** 2021-05-13

**Authors:** Xinlei Zang, Kaige Jin, Feng Zhang

**Affiliations:** Institute of Psychology and Behavior, Henan University, Kaifeng, China

**Keywords:** socioeconomic status, past self, self-evaluation, event-related potential, self-reference

## Abstract

Socioeconomic status (SES) refers to the social position or class according to their material and non-material social resources. We conducted a study with 60 college students to explore whether SES affects past self-evaluation and used event-related potentials (ERPs) in a self-reference task that required participants to judge whether the trait adjectives (positive or negative) describing themselves 5 years ago were appropriate for them. Behavioral data showed that individuals’ positive past self-evaluations were significantly higher than individuals’ negative past self-evaluations, regardless of high or low SES. Individuals with high SES had significantly higher positive past self-evaluations than those with low SES. ERP data showed that in the low SES group, negative adjectives elicited a marginally greater N400 amplitude than positive adjectives; in the high SES group, negative adjectives elicited a greater late positive potential (LPP) amplitude than positive adjectives. N400 is an index of the accessibility of semantic processing, and a larger N400 amplitude reflects less fluent semantic processing. LPP is an index of continuous attention during late processing; the larger LPP amplitude is elicited, the more attention resources are invested. Our results indicated that compared with college students with low SES, the past self-evaluations of college students with high SES were more positive; college students with high SES paid more attention to negative adjectives. However, college students with low SES were marginally less fluent in processing negative adjectives.

## Introduction

In our daily life, we often have conflicting evaluations of our past selves, which are two completely different attitudes. Some scholars proposed a bivariate framework model to explain this phenomenon (Cacioppo et al., 1997). In this framework, individuals can experience complex emotions of happiness and sadness simultaneously (Larsen et al., 2001). Previous studies have shown a separation between negative and positive aspects of the self in adolescents (Ke et al., 2018). In addition, Orchard et al. (2019) used a self-evaluation task to investigate the self-endorsements, and their results found that although depressed adolescents endorsed more negative descriptions of themselves than non-depressed adolescents, positive self-endorsements related to their relationships with other people were not impaired. In other words, depressive symptoms can affect self-evaluation by means of separating positive from negative attitudes.

Our past selves are influenced by many factors related to present selves. According to the temporal self-appraisal theory, people tend to negatively evaluate their past selves (Wilson and Ross, 2001), suggesting that their high self-esteem affects their past self-evaluations. Socioeconomic status (SES) refers to individuals’ or families’ present social position according to their material and non-material social resources. Caregivers’ income, caregivers’ education level, and caregivers’ occupation are often taken as the leading objective indicators for SES measurement (Bradley and Corwyn, 2002). The aim of our study was to examine whether current SES affected past self-evaluation with the use of event-related potentials (ERPs) techniques.

A self-reference task was often employed to investigate temporal self-evaluation. For example, Zhang et al. (2020) adopted a self-reference task to explore the influence of self-relevant information on emotional word processing. They presented participants with a name (three name type: self-name, friend-name, unfamiliar-name), followed immediately by a randomly matched emotional word (three emotional valences: positive, negative, neutral), which did not need for behavioral responses. They recorded the electroencephalography (EEG) data of participants’ perceptions of emotional words. Their results suggested that negative words elicited a smaller N400 amplitude than neutral words. Therefore, they argued that there was a processing advantage for negative words in different stages and the self-reference of a name could affect the cognitive processing of emotional words, which mainly occurred in the late stage of emotional lexical processing. Zhou et al. (2013) required participants to make a judgment of adjectives in positive or negative valences from either self-perspective or other-perspective. Their results showed that smaller N400 amplitudes were elicited by positive stimuli than negative stimuli in the self-perspective condition, but not in the other-perspective condition. Therefore, they claimed a self-positivity bias associated with N400. The self-positivity bias means that individuals pay more attention to positive information relevant to the self, as a positive mood represents the modal experience in healthy people.

The late positive potential (LPP) was used to reflect the process of positive or negative adjectives related to self. When individuals expected positive and negative stimuli, there were neurological differences in their cognition of time, mainly reflected in LPP (Vallet et al., 2019). A study found that the amplitude of LPP induced by self-positive, self-negative, and self-neutral words decreased successively (Herbert et al., 2011). Herbert et al. (2011) requested participants to silently read the pronoun–noun or article–noun pairs (one-third of the nouns from each word category–unpleasant, pleasant, and neutral–were paired with the possessive pronoun “my” or “his” indicating self- or other-reference or the definite article “the” indicating no reference at all) presented on the screen. They argued that there existed a self-positivity bias evidenced by LPP, which reflected the amount of attention individuals took to process emotional words. In addition, Zhang et al. (2020) asked participants to silently read the paired names (self, friend, and unknown) and emotional words (positive, negative, and neutral). They found that negative words elicited a larger LPP amplitude than neutral and positive words. Therefore, they claimed the self-negativity bias in self-referential processing, which needed individuals to continuously pay attention to and deeply encode the negative words relevant to themselves, as the deep processing of negative stimuli related to self could help individuals to avoid threatening stimuli and to better adapt to the environment. Although it is not clear why there are two different self-biases that occurred in the self-reference studies, it has been found that in the experiment of emotional stimulation, the LPP component represents a kind of continuous attention (Solomon et al., 2012), and that the smaller LPP amplitudes are elicited, the less attention to emotional stimuli is paid (James et al., 2018).

To sum up, our study employed a self-reference paradigm (Johnson et al., 2006; D’Argembeau et al., 2008) to explore the neural mechanism of the influence of SES on past self-evaluation. According to previous studies (D’Argembeau et al., 2008; Yang et al., 2020), we chose 5 years ago as the “past.” Given the results that participants with low SES were more likely to experience adverse events and be affected by negative emotions (Waschbusch et al., 2003), we speculated that participants with high SES would pay less attention to negative words, whereas participants with low SES were more likely to be affected by negative words. Therefore, we proposed two hypotheses: (1) compared with the high SES individuals, negative words elicited a smaller N400 amplitude for the low SES individuals; (2) compared with positive words, negative words elicited a larger LPP amplitude for the low SES individuals.

## Materials and Methods

### Participants

Participants with high and low SES were selected through the objective SES scale, which was recompiled (see Appendix 1) based on previous studies (Patro et al., 2011; Gu et al., 2017). In total, 300 SES scales were distributed in a certain university, and 287 completed scales were returned. We calculated the total SES scores by summing the standard *Z*-scores of all items for each participant and then assigned those who ranked in the top 30 into the high SES group (*M*_age_ = 18.87, *SD* = 0.86) and those who ranked in the bottom 30 into the low SES group (*M*_age_ = 19.07, *SD* = 0.92). One participant was excluded from further analysis due to the low signal-to-noise ratio in the EEG signal data. All participants were right-handed and had normal or corrected-to-normal vision. Before the formal experiment, the participants were instructed to complete a pre-experiment to ensure that they understood the procedures. At the end of the experiment, the participants were given 30 CNY as a reward.

### Materials and Stimuli

The stimuli were presented on a 17-inch LCD with a 50 Hz refresh rate and a 1,024 × 768 pixels resolution. The E-prime 2.0 software was used for our experiment and data collection.

The 80 stimulus adjectives consisted of 40 positive and 40 negative trait adjectives (see Appendix 2) from the likableness ratings of 555 personality-trait words (Anderson, 1968). We invited 2 native Chinese fluent in English to translate the 80 English trait adjectives into Chinese.

### Experimental Task and Procedure

The participants were seated in a soundproof, dimly lit room at a distance of about 100 cm from the screen center.

**FIGURE 1:**
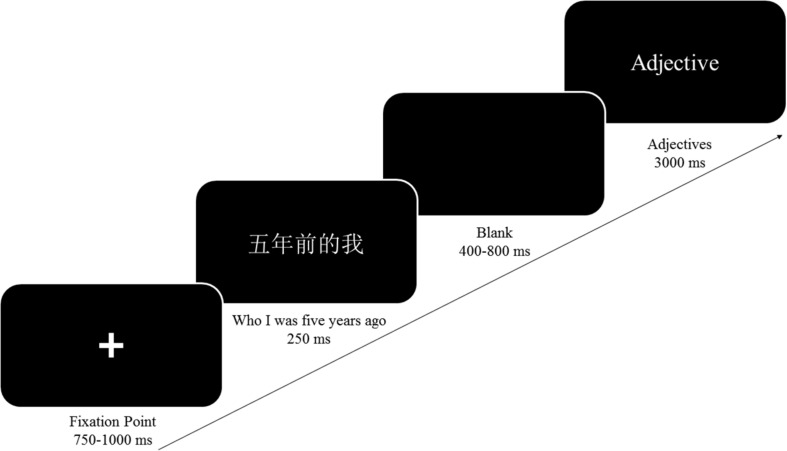
The schematic diagram for typical trials in the self-reference task.

For each trial (see Figure 1), a fixation point was randomly presented on the screen from 750 to 1,000 ms, followed immediately by the presentation of “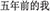 (who I was 5 years ago)” of 250 ms. A stimulus (positive or negative trait adjective) was presented for 3,000 ms after the presentation of a random blank from 400 to 800 ms. When the stimuli were presented, the participants were required to judge whether the trait adjective was appropriate to describe themselves (5 years ago) as quickly as possible. The responses were made by pressing either the “F” or “J” keys on the keyboard. The formal experiment consisted of one block, which included 160 trials (all 80 stimulus adjectives were repeated twice). The order of presentation of the stimuli was completely randomized in our experiment.

### Electrophysiological Data Recording and Analysis

The EEG was collected from two 32-channel BrainAmp DC amplifiers (Brain Products, Gilching, Germany) and recorded from 64 Ag/AgCl electrodes mounted on an electro-cap according to the International 10–20 system. Ground and reference channels were placed on the AFz and FCz electrodes, respectively. An electrode was placed about 2 cm below the right eye to record the electrooculogram (EOG). The EEG signal was digitized at a sampling rate of 500 Hz with a passband of 0.01–100 Hz. Impedances of each electrode were maintained below 5 kΩ.

The EEG data were analyzed offline using the BrainVision Analyzer software (v.2.2). All data were re-referenced offline to averaged bilateral mastoids (TP9, TP10) and were filtered using a 0.3–40 Hz bandwidth. Before segmenting the EEG data, EOG artifacts and electrical noise were rejected manually from the raw data using independent component analysis (ICA). Each EEG epoch was from 200 preceding to 1,000 ms following stimulus onset. Table 1 shows the means and standard deviations of the number of valid trials in each condition after artifact removal. Then, baseline was corrected (200 ms baseline before stimulus). Based on previous studies (Jack et al., 2018; Ogawa and Nittono, 2019; Zhang et al., 2020) and our observation on the grand averaged waveforms, N400 (300–400 ms) and LPP (400–800 ms) were respectively recorded from different electrodes: (Cz, C3, C4) and (Pz, P3, P4, POz, PO3, PO4).

**TABLE 1:** The number of valid trials in different conditions between differing SES groups [*M(SD)*].

	**High SES**	**Low SES**
Positive	76.70 (5.609)	75.24 (11.596)
Negative	76.77 (5.151)	75.79 (11.645)

A two-factor (group × valence) repeated-measure analysis of variance (RM ANOVA) was used to process the mean amplitude of N400 and LPP using the SPSS 25.0 software.

## Results

### Behavioral Data

Table 2 shows the means and standard deviations of behavioral data in each condition.

**TABLE 2:** Descriptive statistics of the proportion choosing Yes and reaction time by participants [*M(SD)*].

		**High SES**	**Low SES**
The proportion of “Yes”	Positive	0.800 (0.197)	0.661 (0.272)
	Negative	0.253 (0.165)	0.320 (0.258)
Reaction time (ms)	Positive	871.745 (270.929)	925.361 (298.584)
	Negative	938.160 (276.078)	975.443 (267.337)

**FIGURE 2:**
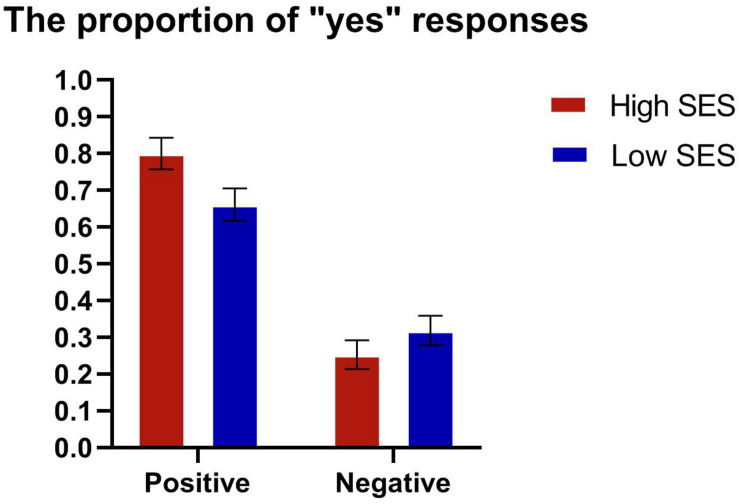
The proportion of YES responses in the self-reference task.

The RM ANOVA for the percentage of “yes” responses of each participant showed that the main effect of valence was significant, *F* (1, 57) = 70.345, *p* < 0.001, η_p_^2^ = 0.552 (see Figure 2). The percentage of “yes” responses in the condition of positive adjectives (*M* = 0.731, *SD* = 0.245) was more than that of negative adjectives (*M* = 0.286, *SD* = 0.216). However, there was no significance of the main effect of group (*p* > 0.10). In addition, the interaction between group and valence was marginally significant, *F* (1, 57) = 3.769, *p* = 0.057, η_p_^2^ = 0.062. Furthermore, the simple effect analysis showed that the percentage of “yes” responses of high SES participants was more than that of “yes” responses of low SES participants (*p* = 0.028) in the condition of positive trait adjectives.

The RM ANOVA for reaction time indicated that the main effect of valence was significant, *F* (1, 57) = 19.924, *p* < 0.001, η_p_^2^ = 0.259. The participants responded faster to positive adjectives (*M* = 898.098, *SD* = 283.675 ms) than to negative adjectives (*M* = 956.486, *SD* = 270.121 ms). However, the main effect of group and interaction between group and valence were both non-significant (*p*s > 0.10).

To sum up, participants with high SES had a more positive attitude toward their past selves than those with low SES, and the reaction time for positive words was shorter than that for negative words, regardless of SES level.

### Electrophysiological Data

**FIGURE 3:**
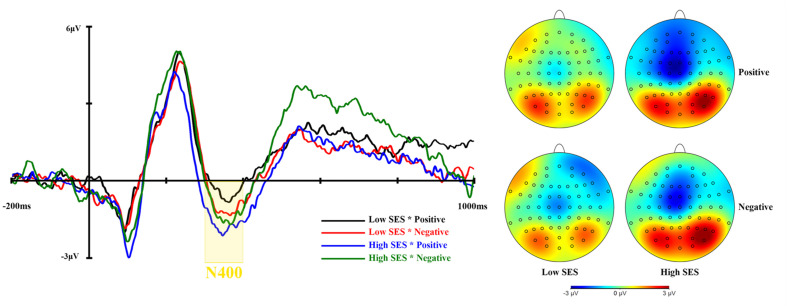
The grand average N400 waveforms of pooling Cz, C3, C4 (left), and the topographic map for each condition (right).

**FIGURE 4:**
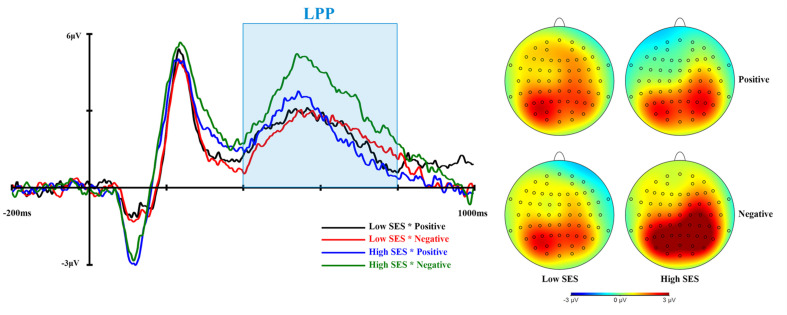
The grand average LPP waveforms of pooling Pz, P3, P4, POz, PO3, PO4 (left), and the topographic map for each condition (right).

Table 3 shows the means and standard deviations of N400 and LPP amplitudes. ERP waveforms and topographic maps of N400 and LPP are shown in Figures 3, 4, respectively.

**TABLE 3:** Descriptive statistics of the ERPs components [*M(SD)*, μV].

		**High SES**	**Low SES**
N400	Positive	−1.739 (3.358)	−0.410 (4.540)
	Negative	−1.229 (3.060)	−0.984 (4.098)
LPP	Positive	2.156 (3.036)	2.116 (2.638)
	Negative	3.464 (3.035)	2.137 (2.849)

#### N400

The RM ANOVA for N400 amplitudes showed that the interaction between group and valence was significant, *F* (1, 57) = 5.803, *p* = 0.019, η_p_^2^ = 0.092. Furthermore, the simple effect analysis indicated that the mean N400 amplitudes in the condition of negative adjectives (*M* = −0.673, *SD* = 4.482) were marginally more negative than those of positive adjectives (*M* = 0.018, *SD* = 5.395) in the low SES group, *p* = 0.053. Moreover, all other simple effects were not significant, *p*s > 0.10. However, there was no significance of two main effects of valence and group (*p*s > 0.10). In other words, negative words elicited a larger N400 amplitude than positive words in the low SES group.

#### LPP

The RM ANOVA for LPP amplitudes showed that a main effect of valence was significant, *F* (1, 57) = 5.819, *p* = 0.019, η_p_^2^ = 0.093. The mean LPP amplitudes in the condition of negative adjectives (*M* = 2.812, *SD* = 2.995) were significantly more positive than those of positive adjectives (*M* = 2.136, *SD* = 2.823). In addition, the interaction between group and valence was significant, *F* (1, 57) = 5.466, *p* = 0.023, η_p_^2^ = 0.088. The simple effect analysis indicated that the mean LPP amplitudes in the condition of negative adjectives (*M* = 3.464, *SD* = 3.035) were significantly more positive than those of positive adjectives (*M* = 2.156, *SD* = 3.036) in the high SES group, *p* = 0.001. Moreover, compared with low SES participants (*M* = 2.137, *SD* = 2.849), those with high SES (*M* = 3.464, *SD* = 3.035) had marginally larger LPP amplitudes in the condition of negative adjectives, *p* = 0.089. Moreover, all other simple effects did not reach significance, *p*s > 0.10. However, there was no significance in the main effect of the group (*p* > 0.10). In other words, negative words elicited a larger LPP amplitude than positive words in the high SES group.

## Discussion

In the present study, we investigated the effects of SES on past self-evaluations using a self-referential paradigm. The behavioral results showed that compared with participants with low SES, participants with high SES endorsed more positive adjectives. In addition, all of the participants responded more quickly to positive words than to negative words regardless of SES. The behavioral results demonstrated that individuals with high SES had more positive self-evaluations significantly in the past than individuals with low SES. Low SES may lead to low self-esteem, which may affect other aspects of individuals as well (Chen et al., 2016). Furthermore, the self-evaluations of positive or negative attitudes were seen as a type of self-esteem (Brown et al., 2001). Therefore, as we expected, individuals with high SES had higher self-esteem for past self and thus had a more positive past self-evaluation. There was a probable reason that individuals with high SES experienced less life stress and family changes in their past lives than those with low SES (Gad and Johnson, 2009), which led to a more positive view of their past selves. It was possible that college students with low SES had been exposed to more negative events during their adolescence.

The ERP results showed that negative words elicited a larger N400 amplitude in the low SES group than positive words. In previous studies, the N400 reflected the process of lexical access (Lau et al., 2008) and lexical-semantic integration (Kutas and Federmeier, 2000), which meant that the reduced N400 elicited by emotional words might indicate fluent semantic processing (Cao and Wang, 2017). Unexpectedly, the N400 results did not support our hypothesis. Although participants with low SES responded more “yes” to positive adjectives than to negative adjectives, the negative adjectives elicited a greater N400 amplitude than the positive adjectives. However, the N400 amplitude had no significant difference between negative and positive adjectives in the high SES participants. This phenomenon may be because college students with low SES were marginally less fluent in processing negative information. The present result was consistent with Waschbusch et al. (2003). They argued that adolescents with anxiety and depression from low SES backgrounds made less helpless attributions for adverse events than adolescents from high SES backgrounds. The N400 was also considered as a component of conflict monitoring, and N400 amplitudes were larger for the semantic conflict (De Pascalis et al., 2009). Therefore, college students with low SES did not think of past themselves as negative, and the larger N400 elicited by negative words was observed.

The ERP data indicated that negative words elicited a larger LPP amplitude in the high SES group than positive words. In previous studies, LPP amplitudes had been seen as an index of late processing needed to pay continuous attention. In the self-appraisals task, negative adjectives elicited a greater LPP amplitude than positive adjectives within the near future self condition (Luo et al., 2013). This result showed that individuals paid more attention to negative adjectives than to positive adjectives, suggesting that individuals had a negative bias. Our study results also showed this negative bias consistent with previous studies (Espuny et al., 2018; Zhang et al., 2020). In addition, we found that the interaction between SES and semantic valence reached significance, and that college students with high SES had a larger LPP amplitude than those with low SES in the condition of negative words. These results indicated that individuals with high SES continuously paid more attention to negative words than those with low SES during late processing. This phenomenon probably occurred because college students with high SES were more afraid of negative information than those with low SES due to a tendency toward perfectionism, and they needed to pay more continuous attention to negative words to alarm themselves. However, compared with college students with high SES, college students with low SES had no self-negativity bias.

Our present study was not without limitations. First, it is necessary to select different samples and consider individual differences in future studies to increase the generalizability of the present findings. Second, given that our study focused on the past self-evaluation as a function of current SES, there was no empirical evaluation within this study of the contrast between past and present self, and thus we will conduct a study to investigate the effects of current SES on both the past self-evaluation and the present self-evaluation, yielding a more comprehensive understanding of SES effect on self-evaluation across time.

In conclusion, college students with high SES reported more positive past self-evaluations and paid more attention to negative words than those with low SES. However, college students with low SES were marginally less fluent in processing negative trait adjectives.

## Data Availability Statement

The original contributions presented in the study are included in the article/Supplementary Material, further inquiries can be directed to the corresponding author.

## Ethics Statement

The studies involving human participants were reviewed and approved by The Ethics Committee of Henan University. The participants provided their written informed consent to participate in this study.

## Author Contributions

FZ and KJ contributed to the conception and design of the study. KJ and XZ were involved in implementing the study, data collection, and data preprocessing. XZ performed the statistical analysis, ERP waveforms, and topographic maps. XZ wrote the first draft of the manuscript. All authors approved the submitted version.

## Conflict of Interest

The authors declare that the research was conducted in the absence of any commercial or financial relationships that could be construed as a potential conflict of interest.
