# Supplementary material for: A Difference of Past Self-Evaluation Between College Students With Low and High Socioeconomic Status: Evidence From Event-Related Potentials
Source: Front Psychol. 2021 May 13;12:629283. doi: 10.3389/fpsyg.2021.629283 (PMC8155721; doi:10.3389/fpsyg.2021.629283)
Supplement: Supplementary file 1 [file Data_Sheet_1.zip › Supplementary material/Appendix 1.docx]

**Appendix 1：The Objective SES Scale**

1.您父亲的受教育水平: ____________

2.您母亲的受教育水平：___________

(1)小学及以下

(2)初中

(3)高中或中专/职高/技校

(4)大学(专科或本科)

(5)研究生及以上

3. 您父亲的职业：____________

4. 您母亲的职业：____________

(1)农业劳动者阶层及非技术、临时工、失业、待业人员，如农民等;

(2)体力劳动工人和个体经营人员、技术工及同级工作者，如建筑工人及相关人员;

(3)一般管理人员与一般专业技术人员、事务性工作人员，包括商业服务业员工阶层、办事人员阶层，如售货员、司机等；

(4)中层管理人员与中层专业技术人员、助理专业人员，包括在各种经济成分的机构( 包括国家机关、党群组织、全民企事业单位、集体企事业单位和各类非公有制经济企业) 中专门从事各种专业性工作和科学技术工作的人员，如教师、医生、技师等；

(5)职业高级管理人员与高级专业技术人员、专业主管人员，包括在党政、事业和社会团体机关单位中行使实际的行政管理职权的领导干部、大中型企业中非业主身份的高中层管理人员和私营企业主阶层，如公务员、公司经理、工头等。

5. 您的家庭(父母等家庭成员总共)月收入是：________元

(1)0~1999

(2)2000~2999

(3)3000~3999

(4)4000~4999

(5)5000~5999

(6)6000~6999

(7)7000~7999

(8)8000~8999

(9)9000~9999

(10)10000~10999

(11)11000~11999

(12)12000及以上
