# Supplementary material for: A Difference of Past Self-Evaluation Between College Students With Low and High Socioeconomic Status: Evidence From Event-Related Potentials
Source: Front Psychol. 2021 May 13;12:629283. doi: 10.3389/fpsyg.2021.629283 (PMC8155721; doi:10.3389/fpsyg.2021.629283)
Supplement: Supplementary file 1 [file Data_Sheet_1.zip › Supplementary material/Appendix 2.docx]

**Appendix 2: Positive and negative trait adjectives**

| 积极形容词 | Positive trait adjectives |
| --- | --- |
| 真诚的 | sincere |
| 正直的 | righteous |
| 体谅的 | considerate |
| 忠诚的 | loyal |
| 诚实的 | honest |
| 聪明的 | intelligent |
| 可靠的 | trustworthy |
| 细心的 | thoughtful |
| 亲切的 | kind |
| 友好的 | friendly |
| 快乐的 | happy |
| 无私的 | unselfish |
| 幽默的 | humorous |
| 负责的 | responsible |
| 信任的 | trustful |
| 宽容的 | broad-minded |
| 愉快的 | pleasant |
| 礼貌的 | polite |
| 有用的 | helpful |
| 热情的 | warm-hearted |
| 能干的 | efficient |
| 警惕的 | alert |
| 机智的 | brilliant |
| 耐心的 | patient |
| 有才的 | talented |
| 合作的 | cooperative |
| 敏锐的 | observant |
| 优雅的 | gentle |
| 守时的 | punctual |
| 明智的 | wise |
| 创造的 | creative |
| 慷慨的 | generous |
| 活力的 | energetic |
| 友善的 | sociable |
| 谦虚的 | humble |
| 自信的 | self-confident |
| 镇静的 | composed |
| 好奇的 | curious |
| 有序的 | orderly |
| 认真的 | earnest |

| 消极形容词 | Negative trait adjectives |
| --- | --- |
| 撒谎的 | liar |
| 虚伪的 | phony |
| 吝啬的 | mean |
| 刻薄的 | cruel |
| 贪婪的 | greedy |
| 自负的 | conceited |
| 粗鲁的 | greedy |
| 自私的 | selfish |
| 敌意的 | hostile |
| 烦人的 | annoying |
| 怀疑的 | distrustful |
| 无礼的 | crude |
| 讨厌的 | unpleasant |
| 嫉妒的 | jealous |
| 偏见的 | prejudiced |
| 胆怯的 | cowardly |
| 冷淡的 | indifferent |
| 懒惰的 | lazy |
| 抱怨的 | complaining |
| 无聊的 | tiresome |
| 厌恶的 | disagreeable |
| 粗心的 | careless |
| 易怒的 | moody |
| 马虎的 | sloppy |
| 专横的 | dominating |
| 幼稚的 | immature |
| 苛求的 | overcritical |
| 奢侈的 | wasteful |
| 悲观的 | pessimistic |
| 愤怒的 | angry |
| 抑郁的 | depressed |
| 迷信的 | superstitious |
| 紧张的 | nervous |
| 笨拙的 | clumsy |
| 悲伤的 | unhappy |
| 犹豫的 | hesitant |
| 羞怯的 | shy |
| 健忘的 | forgetful |
| 沉默的 | silent |
| 孤独的 | lonely |
